# Subacute Arterial Bleeding After Simultaneous Mastopexy and Breast Augmentation with Implants

**Published:** 2018-05

**Authors:** Saulius Vikšraitis, Ernest Zacharevskij, Gytis Baranauskas, Rytis Rimdeika

**Affiliations:** 1 Plastic Surgery Center of Saulius Vikšraitis. Kaunas, Lithuania;; 2 Lithuanian University of Health Sciences Hospital, Kaunas Clinics Plastic and Reconstructive Surgery Department, Kaunas, Lithuania;; 3 Lithuanian University of Health Sciences, Medical Academy, Kaunas, Lithuania

**Keywords:** Breast augmentation, Hematoma, Complication, Esthetic; Breast

## Abstract

Breast augmentation with implants is one of the most commonly performed plastic surgery procedures. The goal of the operation is to increase the size, shape or fullness of the breast. It is accomplished by placing silicone, saline or alternative composite breast implants under the chest muscles, fascia or the mammary gland. This type of operation is no exception concerning the occurrence of complications. The most common early complications include an infectious process, a seroma, and a hematoma, and the late ones are capsular contracture, reoperation, implant removal, breast asymmetry, and rupture or deflation of the implant. The authors present a case of subacute arterial bleeding after simultaneous mastopexy and breast augmentation with silicone implants in a 27-year-old woman. The patient complained of worsening swelling and soreness in the right breast. The patient denied having had any traumas. Ultrasonography indicated 2.5 cm heterogeneous fluid sections around the implant. Therefore, revision surgery was performed, and a hematoma of 650 mL was removed. Hemorrhaging from a branch of an internal mammary artery was found. After the revision, the implant was returned to the lodge. The postoperative period was uneventful. This case report presents a description of a subacute hematoma after simultaneous mastopexy and breast augmentation with silicone implants, which is an extremely rare complication in esthetic surgery.

## INTRODUCTION

Breast augmentation surgery with implants is performed when breasts are small, underdeveloped or wilted after breastfeeding, or for shape improvement in case of asymmetries, in order to increase self-confidence.^1^ Breast shape, size, and appearance is influenced by many factors, the most important of them being age, heredity, weight changes, pregnancy, sun exposure, physical activity, and congenital disease. Breast implants are also used to restore the breast after mastectomy, sex reassignment surgery, etc. Like any other operation, this procedure also has its general risk. However, some complications like bleeding to the lodge of the implant after surgery, infection, fluid accumulation around the implant, capsular contracture, implant rotation, and implant rupture are specific to this procedure.^2^^,^^3^

In this case, subacute spontaneous arterial bleeding occurred. This type of postoperative complication is very rare because after almost 40 years from the first publication of spontaneous bleeding after breast augmentation with implants,^4^ not more than 20 clinical cases have been presented.^5^ This case report presents a description of a subacute hematoma after breast augmentation with silicone implants, which occurred 5 weeks after the surgery.

## CASE REPORT

**Fig. 1:**
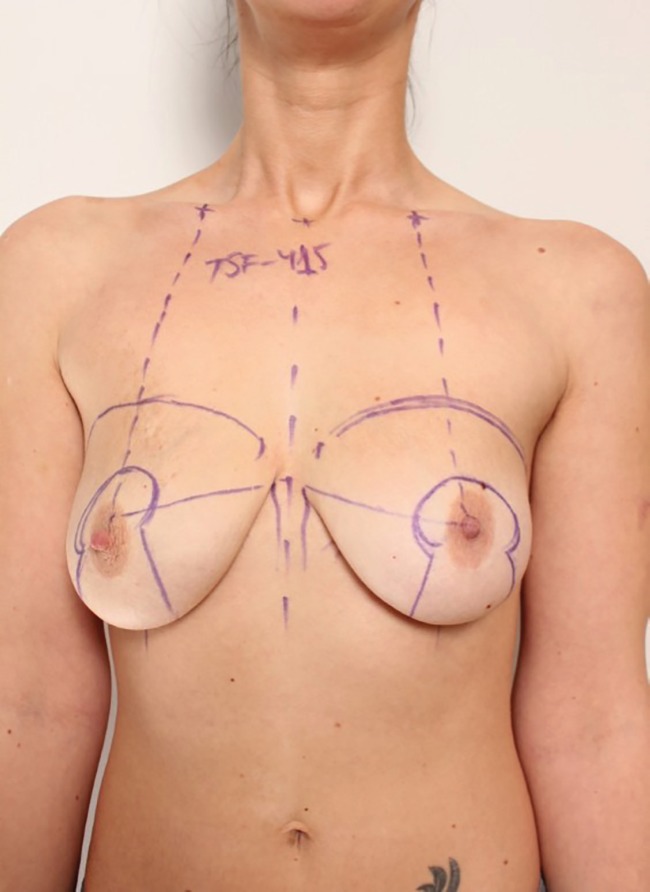
Simultaneous mastopexy according to classical Lejour vertical scar technique and breast augmentation surgery were undertaken using round silicone using implants under the pectoral muscle and a breast lift under general anesthesia

A 27-year-old woman applied for a breast reshaping surgery for aesthetic purposes. There were no other complaints in her medical history. The patient underwent simultaneous mastopexy according to classical Lejour vertical scar technique and breast augmentation surgery using round silicone TSF - 415 mL implants under the pectoral muscle and a breast lift under general anesthesia (Figure 1). During the operation proper hemostasis was achieved using electrocoagulation. Drains were removed next day after the operation with minimum serohemorrhaging fluid volumes. The later postoperative period was also uneventful. 

**Fig. 2:**
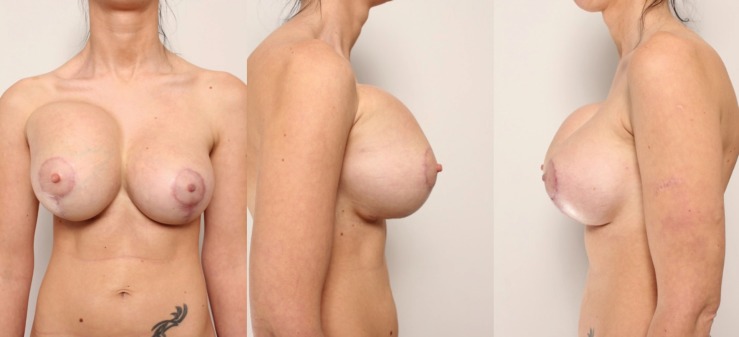
The upper right breast area is significantly swollen and firm.

**Fig. 3:**
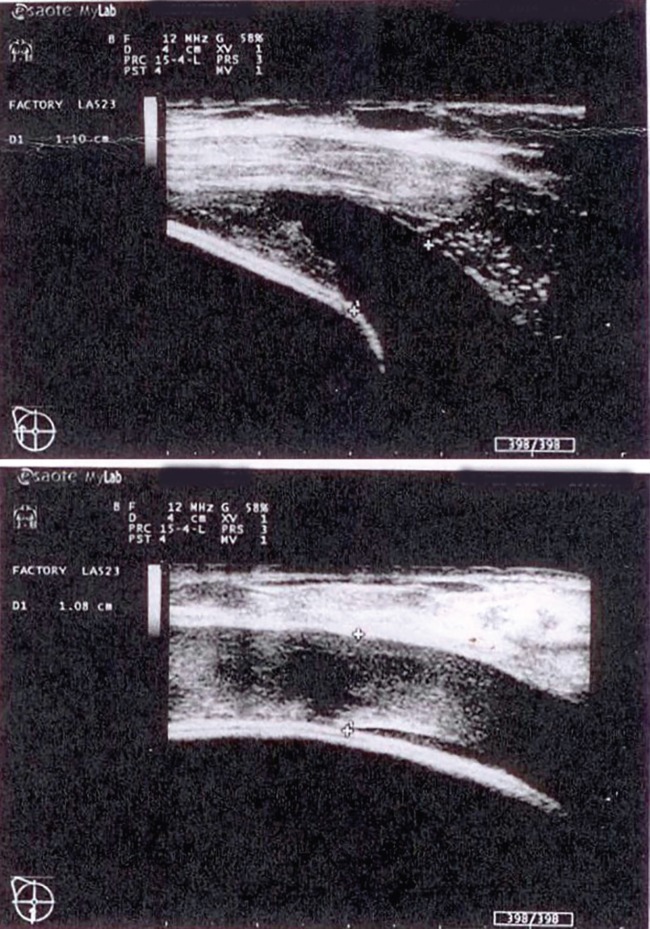
A 2.5 cm heterogeneous liquid strip accumulated around the implant

The patient was discharged from the clinic on the second day after the surgery. Five weeks after the operation patient arrived at the clinic because of tenderness and swelling of the right breast. The patient stated that she had not sustained any traumas. During clinical examination, the upper right breast area was found to be significantly swollen and firm (Figure 2). Ultrasound examination showed a 2.5 cm heterogeneous liquid strip accumulated around the implant (Figure 3). The implant was intact. Complete blood count showed an increased amount of leukocytes, and red blood cells and hemoglobin were at the lower limit of the normal level. 

**Fig. 4:**
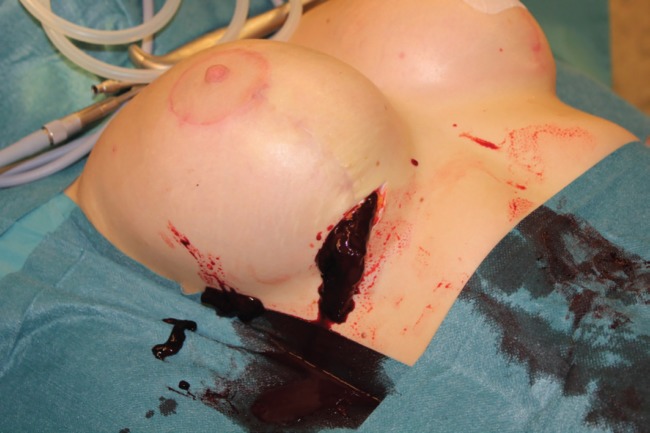
Removal of the fluid and stopping the cause of its accumulation.

**Fig. 5:**
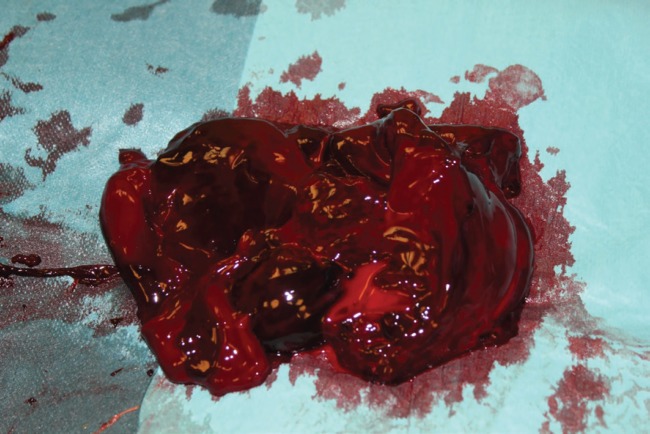
A blood clot of 650 mL was removed.

**Fig. 6:**
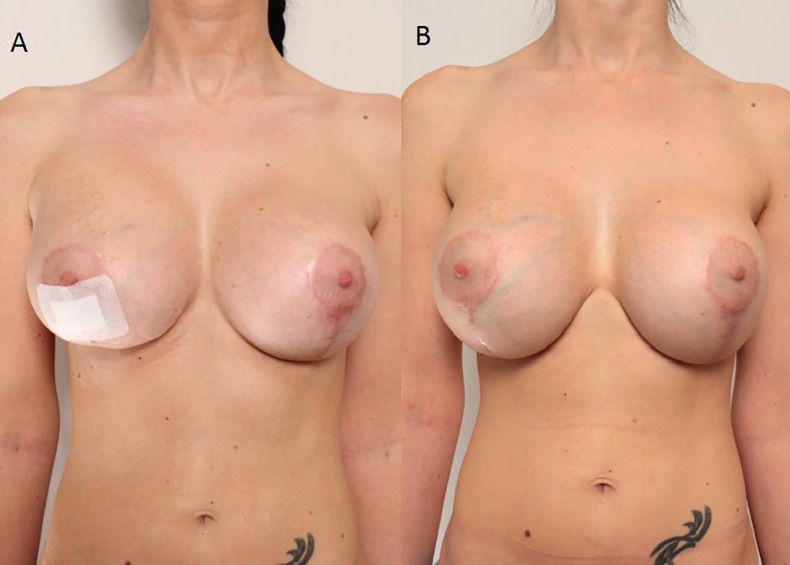
No recurrence of bleeding, no clinical evidence of the implant capsule contracture formation was found.

No coagulopathies were found. The patient was taken to the operating room where she underwent revision surgery. The purpose was to remove the fluid and to find and stop the cause of its accumulation. During the operation (Figure 4), a blood clot of 650 mL was removed (Figure 5). Bleeding from one of the internal mammary artery branches in the implant pocket between the rib cage and the pectoral muscle lower pole was detected and stopped. After the revision, the implant was returned to the lodge. Vacuum drainage was used for one day only. One year after the surgery, there was no recurrence of bleeding, also no clinical evidence of the implant capsule contracture formation was found (Figure 6).

All procedures performed in this study were in accordance with the ethical standards of the institutional and/or national research committee and with the 1964 Helsinki declaration and its later amendments or comparable ethical standards. The patient gave her informed consent prior to her inclusion in this case report. Any details that might disclose the identity of the patient under study were excluded.

## DISCUSSION

Breast augmentation with implants may lead to early or late complications, bleeding being one of the early complications.^6^^,^^7^ Bleeding control is number one priority for any surgeon. Hematoma as a complication of breast enhancement with implants usually occurs within three days after the surgery,^8^ and its incidence is 2% to 10.3% of all breast augmentation operations.^4^^,^^9^^-^^12^ We presented a case of subacute hematoma appearing five weeks after the surgery. Hematomas occurring after breast augmentation are classified into three groups: acute hematomas appear from 3 to 7 days after the surgery, subacute hematomas occur from 7 days up to 3 to 5 months after augmentation, and late hematomas appear 3-5 months or more after mammoplasty.^13^^-^^15^

The cause of acute hematoma after breast augmentation with implants may be inadequate hemostasis during surgery or a congenital, acquired or drug-caused coagulation disorder. Trauma is also one of the factors. The first reported case of late hematoma was published in 1979 by Georgiade *et al*.^4^ Hematoma appeared 2.5 years after the surgery. Authors suggested that the cause of the bleeding was the large dose of corticosteroid used in the saline implant. In 2002, Hsiao *et al*. reported two cases on late hematomas.^10^ The first one occurred two years after the surgery, and the second one- a year postoperatively. 

In both cases, saline-filled, textured silicone prostheses without corticosteroids were used. The cause of the bleeding was only identified in the second patient - neovascularization on the inner surface of the capsule was found. It is thought that mechanical friction between the textured surface of the implant and the high vascular capsule may result in an intracapsular hematoma. In 2009, McArdle and Layt described a case of late hematoma which occurred 12 months postoperatively.^13^ Gel-filled implants were used in this patient. Operative examination showed no signs of bleeding. Supposedly, the subsequent traumatization of the new neovascularized capsule might have been the cause of the late hematoma.^9^

In 2014, Peters *et al*. after examining 5 patients with a late unilateral hematoma hypothesized that bleeding was associated with microfractures in the implant capsule.^14^ The histological analysis of the capsules showed that there was episodic bleeding and reorganization of the hematoma, and the vascular spasm of the damaged vessel was impossible because of the rigidity of the capsule. Late hematomas after breast augmentation with implants also occur because of a sudden rupture of the implant and the capsule, usually – due to the effect of an external force. An increasing chronic hematoma may appear when chronic inflammatory processes are active, the friction force between the surface of the implant and capsule is high, manifestations of coagulopathies are seen, there is a probability of capillary damage because of the rigidity of the capsule, or steroids are used.^14^

Nasr *et al*. reported a case of a subacute hematoma that developed three weeks after the augmentation mammoplasty.^15^ In this case, mild physical stress was sufficient to trigger bleeding from a branch of the internal mammary artery because it was eroded by the friction between the implant and the vessel. In our case, the cause that triggered bleeding was unknown. Patient denied trauma, use of anticoagulants or history of any coagulopathies. Classical Lejour vertical scar technique for the breast lifting affects skin, subcutaneous fat and glandular tissues, but there is no influence to the pectoral muscle and its vascularisation.^16^

In our case no suspension sutures were performed to the underneath muscle. Damaged bleeding artery was found in the implant pocket under the muscle in the border where lower internal pectoral muscle pole attaches to the ribs and sternum. If the artery was on tension even a slight displacement of the implant in condition of a scar formation could have made damage. Also this theory makes sense with big and high profile silicone implants because of the mechanical tension to the muscle and other tissues. We believe that the treatment tactics for acute and subacute hematomas after breast augmentation with implants should be revision surgery– an inframammary incision with subsequent evacuation of the hematoma, followed by the identification and elimination of the source of bleeding. 

A bacterial culture test is useful for prophylactic antibiotic therapy after surgery. Drainage for reducing the risk for complications should be carried out.^17^ As for late hematoma, the procedure is the same, except for the histological examination of the capsule tissue; replacement of the implant is questionable. Subacute hematoma after breast augmentation with implants is an extremely rare complication in esthetic surgery. Method of breast augmentation and mastopexy could be one of the reasons for bleeding. History of trauma or use of specific pharmaceuticals also could not be ruled out. But most of the times the etiology of bleeding is unknown. This type of complication requires surgical treatment. We recommend that each case of late and subacute hematomas after breast augmentation with implants should be described and published for the scientific evaluation of this complication.
